# Modified Masquelet Technique Using Allogeneic Graft for a Gustilo-Anderson Type III-A Open Fracture of the Femur with an 8 cm Bone Defect

**DOI:** 10.1155/2021/8829158

**Published:** 2021-03-10

**Authors:** Hichem Issaoui, Mohammed Reda Fekhaoui, Moheddin Jamous, Alain-Charles Masquelet

**Affiliations:** ^1^Department of Orthopedic Surgery and Trauma, Regional Hospital Center of Orleans, Orleans, France; ^2^Department of Trauma and Orthopedic Surgery, Ibn Sina University Hospital, Faculty of Medicine, Mohammed V University of Rabat, Rabat, Morocco; ^3^Department of Orthopedic Surgery and Trauma, Hospital Center Avallon, Auxerre, France; ^4^Department of Orthopedic Surgery and Trauma, Hospital Saint-Antoine, Paris, France

## Abstract

The induced membrane technique was initially described by Masquelet et al. in 1986 as a treatment for tibia nonunion; then, it became one of the established methods in the management of bone defects. Several changes have been made to this technique and have been used in different contexts and different methodologies. We present the case of a 16-year-old girl admitted to our department for a polytrauma after a motorcycle accident. She presented a Gustilo III-A open fracture of the right femoral shaft with a large bone defect of 8 centimeters that we treated with a modified Masquelet technique. In the first stage, an Open Reduction and Internal Fixation of the fracture was made using a 4,5 mm Dynamic Compression Plate and a PMMA cement was inserted at the bone defect area. The second stage was done after 11 weeks, and the defect area was filled exclusively with bone allograft from a bone bank. Complete bony union was seen at 60 weeks of follow-up. After the removal of the implants by another surgeon, the patient presented an atraumatic fracture of the neoformed bone that we treated with intramedullary femoral nailing associated with a local autograft using reaming debris. A complete bony union was achieved after 12 weeks with a complete range of motion of the hip and knee. The stability given to the fracture is essential because it influences the quality of the induced membrane and Masquelet has recommended high initial fixation rigidity to promote incorporation of the graft. It is recommended to delay the second stage of this technique after 8 weeks, especially in femoral reconstruction, to optimize the quality of the induced membrane. Several studies used a modified induced membrane technique to recreate a traumatic large bone defect, and all of them used an autologous bone graft alone or an enriched bone graft. In this case, the use of allograft exclusively seems to be as successful as an autologous or enriched bone graft. Now, with the advent of bone banks, it is possible to get an unlimited amount of allograft, so additional research and large studies are necessary before giving recommendations.

## 1. Introduction

Gustilo-Anderson type III open fracture is defined as an open fracture with extensive soft tissue damage and bone loss [1]. These fractures usually occur after a high-energy trauma with a risk of infection approaching 7% [2]. The treatment remains a challenge for most orthopedic surgeons. Conventional treatment strategy of Gustilo type III injury involves initial debridement to minimize infection followed by flap surgery and bone defect reconstruction [3]. A critical bone loss is defined by a defect exceeding 2-2,5 times the diameter of the affected bone [4]. Various techniques have been described to reconstruct bone defects such as transfer of the vascular fibula, segmental bone transport, free vascular or nonvascular fibula graft, autogenous graft, and megaprothesis [5]. The induced membrane technique was reported by Masquelet et al. in 1986 [6] and described as two-stage reconstruction of bone loss. It is a widely used technique supported by several publications with very good outcome and a high success rate. In this case report, we used a modified Masquelet technique to reconstruct a grade IV-B femoral bone loss (8 centimeters) in a Gustilo-Anderson III-A open fracture.

## 2. Case Presentation

**Figure 1:**
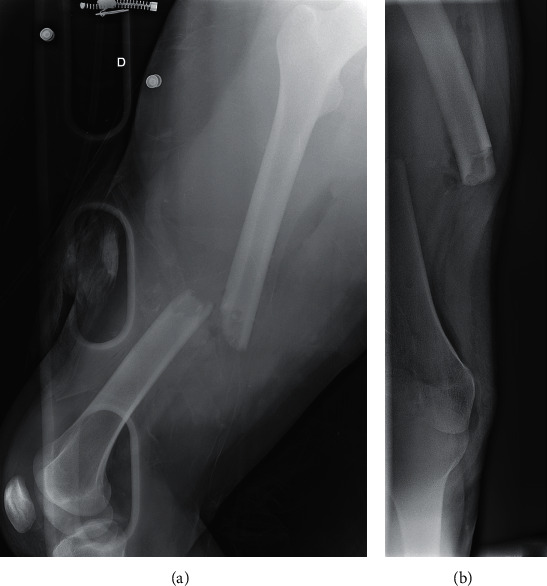
Anteroposterior (a) and lateral (b) X-rays of a Gustilo-Anderson III-A open fracture of the femur in a 16-year-old female patient.

**Figure 2:**
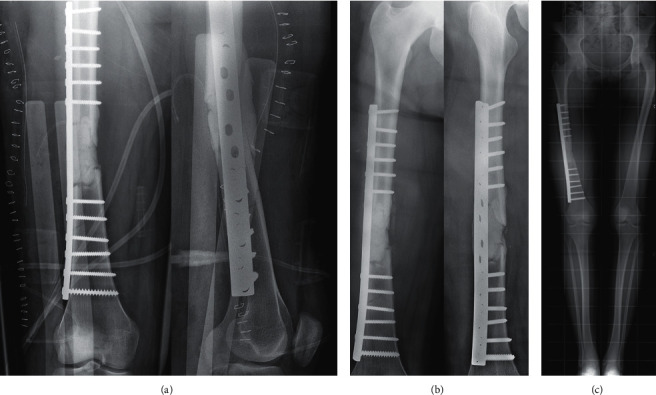
After a surgical debridement comes the first stage of the modified Masquelet technique. (a) The large defect was filled with cement, and the fracture was stabilized with a 4,5 mm Dynamic Compression Plate. (b) Follow-up views at 4 weeks post first stage. (c) At the full-length weight-bearing (FLWB) X-ray, there was no leg length discrepancy.

**Figure 3:**
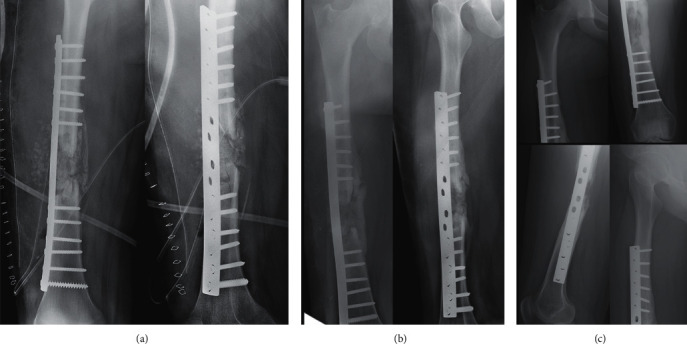
Second stage of the modified Masquelet technique with removal of the cement and the insertion of allograft from a bone bank (a). Succeeding follow-up views at 12 weeks (b) and 32 weeks (c).

**Figure 4:**
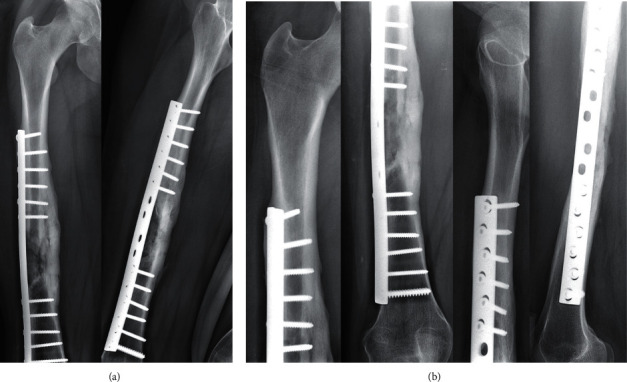
Complete bony union with cortical reconstitution and full integration of the allograft at 60 weeks of follow-up (a). The return to sports activities was authorized after 84 weeks (b).

**Figure 5:**
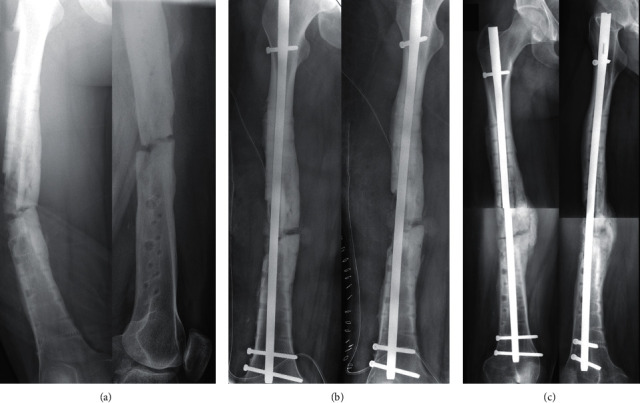
The patient presented an atraumatic fracture of the neoformed bone (a) after the removal of the implants 32 weeks after the bone healing. We opted for an intramedullary femoral nailing associated with a local autograft using the reaming debris (b). Complete bony union was achieved at 12 weeks of follow-up (c).

**Figure 6:**
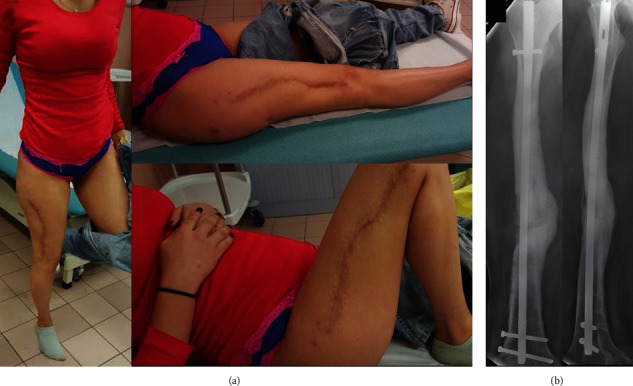
Final clinical (a) and radiological result at 32 weeks of follow-up from the atraumatic fracture (b).

A 16-year-old girl was admitted to our department for a polytrauma after a motorcycle accident. After initial management in the prehospital phase, the patient was hemodynamically stable and presented a Gustilo III-A open fracture of the right femoral shaft (32A3-C of AO/OTA classification) [7] with a large bone defect of 8 centimeters caused by the trauma (grade IV-B according to Winquist-Hansen modified classification; Table 1) (Figure 1) [8]. The patient also presented a traumatic brain injury with loss of consciousness for a few seconds without any abnormalities on head CT, a 7 cm wound of the scalp and the temporal region, an occipital condylar fracture with a C1-C2 diastasis, and a superior and inferior pubic rami fracture. A cervical spine immobilization was first done using a hard collar; then, the protocol for open fractures began in the emergency department: two grams of amoxicillin-clavulanic acid were administered with a tetanus toxoid booster, the wound was covered with a sterile dressing, the fracture was immobilized, and the patient was shifted to the operating room. A surgical debridement and wound irrigation were done; then, the first stage of the modified Masquelet technique was performed. An Open Reduction and Internal Fixation of the fracture was made using a 4,5 mm Dynamic Compression Plate (16 holes), and a PMMA cement was inserted at the bone defect area to act as a spacer and help the production of a membrane (Figure 2). Before the second stage, clinical signs of infection were excluded and biological exams (complete blood count, C-reactive protein, and erythrocyte sedimentation rate) were normal. As follows, the second stage was done after 11 weeks. The membrane was opened, and the cement was removed; next, permeabilization of the medullary canal was done with a curette at both bone extremities to remove interposed fibrous tissue and in order to restart the vascularization. The defect area was filled with bone allograft from a bone bank (Figure 3(a)). At 12 weeks from the second stage, bone callus starts to form on the medial aspect of the fracture (Figure 3(b)), so a partial weight-bearing was initiated; then, a protected weight-bearing was done at 32 weeks after the surgery (Figure 3(c)). Complete bony union with cortical reconstitution and full integration of the allograft was seen at 60 weeks of follow-up (Figure 4(a)). The full weight-bearing was permitted, and the return to sports activities was authorized after 84 weeks (Figure 4(b)). Upon patients' request, the removal of the implants was done by another surgeon 32 weeks after the bone healing (92 weeks after the second stage of the modified Masquelet technique). It was early, in our opinion, so a few weeks later, the patient presented an atraumatic fracture of the neoformed bone (Figure 5(a)). This time, we opted for an intramedullary femoral nailing associated with a local autograft using the reaming debris (Figure 5(b)). A full weight-bearing was initiated after 12 weeks when a complete bony union was achieved (Figure 5(c)). The final clinical and radiological result at 32 weeks of follow-up was excellent with a complete range of motion of the hip and knee and a complete bone remodeling (Figure 6).

## 3. Discussion

Traumatic segmental bone defects of the femur are uncommon. Secondary reconstructions after maintaining the bone defect, just as it is, remain very difficult because of the fibrosis and the risk of involvement of major vascular and nervous structures in this tissue [6]. An autogenous cancellous bone graft is considered as the “gold standard” in the management of bone defects that does not exceed the diameter of the affected bone [6]. Bone shortening is possible when the bone defect is lesser than 3 centimeters. However, as the volume of the bone defect increases, the more difficult the treatment becomes. The induced membrane technique was initially described by Masquelet et al. in 1986 as a treatment for tibia nonunion [6, 9]. Later, this technique became one of the established methods in the management of bone defects, supported by several publications with very good outcome and a high success rate [10]. Several changes have been made to this technique and have been used in different contexts and different methodologies [11]. The procedure consists of two stages [6]. In the first stage, a PMMA cement is planted in the zone of bone defect. It is acting as a spacer (to maintain the length of the bone) and facilitates the formation of a membrane which contains osteoprogenitor cells and has the aptitude to release inductive molecules capable of stimulating osteogenesis [12, 13]. Additives included to the cement, such as antibiotics, can disturb the formation of the membrane (thickness and quality) and have an effect on bone healing [14]. The second stage is initiated after 6 to 8 weeks when the secretion of growth factors reaches his top [12]. Masquelet et al. recommend, in the femoral reconstruction, to delay the second phase until the third month given the presence of a single vascular axis (the femoral artery) which reduces the delay of vascularization of the induced membrane (circumferential vascularization). An early second stage can be risky because the induced membrane is fragile, poorly vascularized, and can be a source of bone graft damage and loss [6]. In our case, the second stage was done after 11 weeks, which is acceptable. The membrane is opened, the cement is removed, and the defect area is filled with autogenous cancellous bone graft harvested from the iliac crest. The induced membrane left in place prevents the resorption of the graft and promotes osteoinduction. A bone substitute (demineralized allograft) can be added to an insufficient amount of autograft in a ratio that does not exceed 1 : 3 [12]. In this case, we used a modified Masquelet technique in which the large 8 centimeters of bone defect was filled only with an allograft from a bone bank. Several studies reported in the literature embraced a modified induced membrane technique to recreate a traumatic large bone defect, and all of them used an autologous bone graft alone or an enriched bone graft (autologous bone graft with allograft, osteoprogenitor cells, growth factors, platelet-rich plasma, and bone marrow concentrate aspirate) [11, 15–17]. The use of a modified Masquelet technique with a pure allograft is very rare. Bone callus starts to form at 12 weeks from the second stage, and a complete bony union was seen at 60 weeks of follow-up. This delay of consolidation is acceptable and coherent with the literature [11, 18]. The stability given to the fracture is essential because the persistence of mobility induces an inflammatory reaction that influences the quality of the induced membrane [6]. External fixation is known to provide relative fracture stability. On the other hand, intramedullary nailing makes more difficult the removal of the cement and the preservation of the induced membrane [6]. Masquelet et al. have recommended high initial fixation rigidity to promote incorporation followed by more flexible fixation to promote remodeling and cortication [19]. In our case, we choose an initial internal fixation with a rigid plate to assure absolute stability of the fracture, to optimize the quality of the induced membrane, to allow early joint mobility, and to simplify the second stage of the procedure. Thereafter, the occurrence of an atraumatic fracture does not decrease in any way the quality of the technique and the newly formed bone; it is secondary to a premature plate removal, especially since the risk of a microfracture near a screw hole is always present [20]. Getting a complete bone union, 12 weeks after the femoral nailing, confirms that the modified Masquelet technique guarantees the growth of a newly formed bone of good quality, while underlining the fact that we used allograft exclusively.

## 4. Conclusion

The modified Masquelet technique is a simple, reliable, and reproducible technique that can be used for reconstruction of large bone defects with a time to union that does not depend on the size of the defect unlike distraction osteogenesis. Initial internal fixation with a rigid plate is, in our opinion, the reasonable choice to obtain absolute stability. It is recommended to delay the second stage of this technique after 8 weeks, especially in femoral reconstruction, to optimize the quality of the induced membrane. The use of allograft exclusively seems to be as successful as an autologous or enriched bone graft. Now, with the advent of bone banks, it is possible to get an unlimited amount of allograft, so additional research and large studies are necessary before giving recommendations.

## Figures and Tables

**Table 1:** Winquist-Hansen modified classification [8].

Grade I	No or minimal comminution
Grade II	More than 50% contact between the two fragments, moderate comminution
Grade III	Less than 50% contact between the two fragments, moderate to severe comminution
Grade IV	Severe comminution with no contact between the fragments and segmental bone loss less than 5 cm (IV-A) or more than 5 cm (IV-B)
